# Excerpts

**Published:** 1890-06

**Authors:** John A. Miller

**Affiliations:** Professor of Medical Chemistry and Toxicology in the Medical Department of Niagara University


### EXCERPTS.

BY JOHN A. MILLER, M. A., PH. D.

Professor of Medical Chemistry and Toxicology in the Medical Department of Niagara University.

REDUCING SUBSTANCES IN URINE. By H. H. Ashdown. (*Brit. Med. J.*, 1890, i. 169; *J. Chem. Soc.*, March, 1890, pp. 279.) *Glycuronic Acid* reduces Fehling's solution, and is therefore apt to be mistaken for sugar in urine. It can only be identified with certainty by isolating it and examining its properties. A ready distinction, however, between sugar and this substance is that the addition of yeast to the former, even when dissolved in the urine, causes the occurrence of the alcoholic fermentation, and to the latter does not.

The diagnosis of diabetes, in man, must in the future be more carefully made, as in one case the reducing substance was found to be wholly glycuronic acid ; the man in whom this occurred is in perfect health, and no symptoms of diabetes are present. As this is the only case of the kind recorded, it will be a question of great importance in relation to life insurance to ascertain whether this state of things occurs in other cases.

In animals the appearance of glycuronic acid in the urine is readily produced by the administration of certain drugs, camphor, phenol, etc.

In the author's research the following experiments were made :

The urine secreted after drugging with morphine, contains not sugar, but glycuronic acid; after the administration of chloroform, glycuronic acid, and not sugar, is present. The so-called glycosuria of curare poisoning is not due to the presence of sugar, as there is no fermentation with yeast. The administration of ether does not cause the appearance of any reducing substance in the urine. After section of the renal nerves, a paralytic secretion occurs. This contains a reducing substance, which was found to be glycuronic acid.

NITROGENOUS CONSTITUENTS OF HUMAN URINE. By E. Schultze. (*Pflueger's Archiv.*, 45-401 ; *J. Chem. Soc.*, March, 1890, pp. 280.) The investigation was carried out on a human subject, its object being to compare the variations in the amount of urea-nitrogen, and the nonurea-nitrogen in relation to diet. The conclusions arrived at are, viz. :

1. The urea-nitrogen increases in proportion to total nitrogen as the diet approaches a purely albuminous composition.

2. The uric acid increases absolutely, but diminishes relatively, both to total nitrogen and to the urea on a meat diet, especially if large quantities of alkaline water be taken, and alcoholic drinks and narcotics be avoided.

3. Probably in fever the same relation of uric acid to total nitrogen and urea holds.

4. The use of abundant quantities of water and withdrawal of alcoholic beverages in cases of gout has thus a scientific basis.

EFFECT OF FEEDING ON THE SECRETION OF AMIDIC SUBSTANCES. By E. Schultze. (*Bied. Centr.* 18-733 ; *J. Chem. Soc.*, March, 1890, pp. 278.) The results of the author's experiments are, viz. : (1.) The nearer the feeding of persons approaches to a pure animal diet the greater is the amount of nitrogen as urea, in proportion to the total nitrogen of the urine. (2.) The relation of uric acid to the total nitrogen decreases with meat diet, as opposed to feeding with mixed food. This occurs in a still greater degree with a meat diet, with use of abundance of alkaline water and absence of alcohol and narcotics, although the absolute amount of uric acid increases. (3.) The same holds good with regard to the relation of uric acid to urea. (4.) It is very probable that in fever, even in absence of respiratory derangement, there is not only an absolutely larger amount of uric acid produced, but also an increased proportion of uric acid relatively to total nitrogen and to urea. (5.) The use of plenty of alkaline water, and the disuse of alcohol in the treatment of gout, are justified by experimental evidence which cannot be denied; these factors seem to be of greater importance than the prohibition of meat.

Action of Related Chemical Compounds on Animals. By W. Gibbs and H. A. Hare. (*Amer. Chem. J.*, 11-435; *J. Chem. Soc.*, March, 1890, pp. 280.) The authors publish the first part of a research, having for its object a systematic study of the relation between the chemical constitution of compounds and their action on the animal organism. Dogs and frogs were used in these experiments, and a description is given of the action of the nitrophenols, the nitranilines, and the amido- and nitro-benzoic acids, when administered by the stomach or hypodermically.

The nitro-phenols cause death by paralyzing the heart, and not by a respiratory action; the nervous system is unaffected by them, except that the vagus nerves are slightly stimulated by the ortho- and meta-compounds, but depressed by the para-compound. The lethal dose per kilo. of body-weight is 0.1 gram. of the ortho-, about 0.1 of the meta-, or 0.01 gram of the para-compound, when injected into the jugular vein. The nitranilines all act by stimulating the peripheral vagi, and so producing a very marked slowing of the pulse. The lethal dose in the case of the ortho-compound is 0-3 grams per kilo.; methemoglobin is produced in the blood, and the sensory side of the spinal cord is slightly affected, but this is probably caused indirectly by the changes in the blood. Given by the stomach it produces curious paroxysms of sneezing. The meta-compound has a very feeble effect on the nerves, and this depends on the development of methemoglobin in the blood, all the symptoms being those of aniline poisoning. The para-compound is the most poisonous, the lethal dose being 0.04 gram. per kilo. of body-weight when injected into the jugular vein. The amido-benzoic acids and the nitro-benzoic acids were found to be without effect on the animal organism.

